# Editorial: Food Proteomes: Beyond Their Nutritional Value

**DOI:** 10.3389/fnut.2021.744473

**Published:** 2021-08-24

**Authors:** Xavier Gallart-Palau, Xinya Hemu, Maria-José Motilva, Aida Serra

**Affiliations:** ^1^University Hospital Institut Pere Mata, Reus, Spain; ^2^Neuroscience Division, Institut Investigació Sanitària Pere Virgili (IISPV), Reus, Spain; ^3^Centro de Investigación Biomédica en Salud Mental (CIBERSAM), Instituto de Salud Carlos III (ISCIII), Madrid, Spain; ^4^Lleida Institute for Biomedical Research Dr. Pifarré Foundation – IRBLleida, +Pec Proteomics Research Group, University of Lleida- UdL, Lleida, Spain; ^5^Proteored – Instituto de Salud Carlos III (ISCIII), Madrid, Spain; ^6^School of Biological Sciences, Nanyang Technological University, Singapore, Singapore; ^7^Spanish National Research Council (CSIC) Instituto de Ciencias de la Vid y del Vino (ICVV), Logroño, Spain; ^8^IMDEA-Food Research Institute, +Pec Proteomics, Campus of International Excellence UAM+CSIC, Old Cantoblanco Hospital, Madrid, Spain

**Keywords:** dietary proteomes, food proteins, milk, human milk, breastfeeding, human milk substitutes, proteolysis, foodomics

This Research Topic collects diverse studies focused on the in-depth characterization of dietary proteomes by evaluating their singular features, possible roles in health and disease conditions; as well as exploring the changes caused by industrial processing to food proteomes. Of note, characterization of dietary proteomes has been performed by using high throughput mass spectrometry strategies in all compiled studies.

The first article of this topic entitled “Structural Changes and Evolution of Peptides During Chill Storage of Pork” (Zou et al.) is focused on the investigation of post-mortem aging of dietary proteins in fresh pork meat (*Longissimus dorsi*) during chill-storage and their relevance in protein digestibility. The authors reported that protein denaturation and unfolding occurs in <3 days of chill-storage. The ordered and stable structures of meat proteins were gradually destabilized during chill-storage to become a mesh of loose and disordered protein fragments due to the action of endogenous enzymes. This basal proteolysis increased the number of exposed tyrosine and tryptophan residues as well as the number of exposed digestion sites. Myofibrillar proteins followed by sarcoplasmic proteins were the families of proteins more degraded in meat during chill-storage. Authors also demonstrated how the new hydrophobic status and the altered protein structure increases protein digestibility. Proteomics characterization of proteolytic peptides demonstrated that although long storage times led to degradation of proteins into amino acids or small peptides, moderate chill storage increased the production of antioxidant, ACE inhibitors and DPP-IV inhibitory bioactive peptides.

Moreover, it has to be emphasized that human milk (HM) is the optimal milk source for newborns due to its nutritional composition and non-nutritive bioactive fraction, specially when they are born prematurely (1). HM composition is highly complex, and although milk proteome only represents around 0.9–1.2 g/dL (1), this fraction is not only considered a macronutrient fraction, as it is rich in innate-immune proteins that contribute to the development of the infant's innate immunity (2). In this line, Sari et al. performed a comparative study of human milk proteomes obtained from donors from eight cities of China during the first 6 month of lactation as part of the Chinese Human Milk Project. This study entitled “Comparative proteomics of human milk from eight cities in China during 6 months of lactation in the Chinese Human Milk Project (CHMP) study” (Sari et al.) reinforces the fact that HM composition is influenced by endogenous and exogenous factors. In this proteomics comparative study Sari et al. reported differences in the HM proteomes across individuals and along the lactation period depending on the geographic localization. As proposed by the authors these findings account for a potential dynamic mechanism that may fulfill the changing infant needs alongside their development. The geographic differences observed by the authors were mainly explained by 12 immune-related proteins, including different lactalbumins, lactoferrins, and immunoglobulins. These differences may be the response toward the pathogen pressure of the environment [(3), Sari et al.]. In-depth analysis of differentially expressed proteins also demonstrated their close relation with infant host defense. Remarkably, a significant subset of triglycerides metabolic process-associated proteins were differentially enriched depending on the location between the first and fifth month of lactation. Nonetheless, authors indicate that the global differences observed in HM compositions along the lactation period were the result of a more complex and subtle modulation mechanism mediated by a higher number of proteins.

When HM is not an option, the selection of a good HM substitute with adequate nutritional quality is crucial for the newborn development. In this line, the other two studies compiled in this Research Topic investigate the composition and characteristics of milk proteomes from different species. Chopra et al. with the study entitled “High-Resolution Mass Spectrometer–Based Ultra-Deep Profile of Milk Whey Proteome in Indian Zebu (Sahiwal) Cattle” reported the first in-depth characterization of the low abundant proteins fraction present in milk whey of indian zebu (Sahiwal) cattle, being sahiwal a more disease and heat resistant cattle compared to most farmed *Bous taurus* cattle. In this study Chopra et al. optimized the protein extraction procedure and by combining different in gel and in solution strategies identified more than 6200 proteins from bovine milk whey (Chopra et al.). A high proportion of these low abundant proteins were found to display an immune regulation and host defense role in bovine. This subset of proteins included lactoglobulins, lactoperoxidases, caseins, and other immune-related proteins such as complement C3. The chromosomal mapping of the identified proteins demonstrated an uneven contribution of all chromosomes in the translation of the identified bovine whey proteome.

Continuing with the search of mammalian milks with high similitudes with HM, Zhang et al. in the study entitled “Quantitative Label-Free Proteomic Analysis of Milk Fat Globule Membrane in Donkey and Human Milk,” included in this Research Topic, characterized the specific and crucial fraction of milk fat globule membrane (MFGM) proteins of donkey milk (DM) (Zhang et al.). It is known that DM is a mammalian milk that highly resembles HM as it displays similar protein and lactose contents, and fatty acids and protein profiles compared to HM (4). Additionally, MFGM proteins of DM are smaller and therefore show high digestibility in infants (5). Due to these reasons, the interest in DM from the dairy and infants-food industries is growing. The detailed characterization of MFGM performed by Zhang et al. demonstrated the significant presence of common MFGM proteins in both analyzed milks, representing the 43% of the total MFGM proteome in DM. Besides, the fraction of uniquely identified proteins from DM included proteins such as semaphoring 7A, complement C3, proteins from the solute carrier (SLC) superfamily or multiple apolipoproteins (Apos), among others. Similarly to what was reported by Chopra et al. for sahiwal bovine milk (Chopra et al.), the gene ontology and KEGG pathway enrichment analysis demonstrated that the uniquely identified MFGM proteins from DM were involved in immune response, participating in complement activation, defense response and positive regulation of B cell activation, fact that may be positive when using DM in infant formulas.

With all this, we believe that this Research Topic puts together a representative collection of studies that reflect the state of the art in this interesting and emerging field aimed to decipher the secrets and biological role(s) of dietary proteomes and their potential implications in health and disease conditions.

## Author Contributions

All authors listed have made a substantial, direct and intellectual contribution to the work, and approved it for publication.

## Conflict of Interest

The authors declare that the research was conducted in the absence of any commercial or financial relationships that could be construed as a potential conflict of interest.

## Publisher's Note

All claims expressed in this article are solely those of the authors and do not necessarily represent those of their affiliated organizations, or those of the publisher, the editors and the reviewers. Any product that may be evaluated in this article, or claim that may be made by its manufacturer, is not guaranteed or endorsed by the publisher.
